# Analysis of the Co-existence of Long-range Transport Biomass Burning and Dust in the Subtropical West Pacific Region

**DOI:** 10.1038/s41598-018-27129-2

**Published:** 2018-06-12

**Authors:** Xinyi Dong, Joshua S. Fu, Kan Huang, Neng-Huei Lin, Sheng-Hsiang Wang, Cheng-En Yang

**Affiliations:** 1 Department of Civil and Environmental Engineering, the University of Tennessee, Knoxville, TN 37996 USA; 2 Climate Change Science Institute, Oak Ridge National Laboratory, Oak Ridge, TN 37831 USA; 3 Center for Atmospheric Chemistry Study, Department of Environmental Science and Engineering, Fudan University, Shanghai, 200433 China; 4 Department of Atmospheric Sciences, National Central University, Chung-Li, 32056 Taiwan

## Abstract

Biomass burning and wind-blown dust has been well investigated during the past decade regarding their impacts on environment, but their co-existence hasn’t been recognized because they usually occur in different locations and episodes. In this study we reveal the unique co-existence condition that dust from the Taklamakan and Gobi Desert (TGD) and biomass burning from Peninsular Southeast Asia (PSEA) can reach to the west Pacific region simultaneously in boreal spring (March and April). The upper level trough at 700hPa along east coast of China favors the large scale subsidence of TGD dust while it travels southeastwards, and drives the PSEA biomass burning plume carried by the westerlies at 3–5 km to descend rapidly to around 1.5 km and mix with dust around southeast China and Taiwan. As compared to the monthly averages in March and April, surface observations suggested that concentrations of PM_10_, PM_2.5_, O_3_, and CO were 69%, 37%, 20%, and 18% higher respectively during the 10 identified co-existence events which usually lasted for 2–3 days. Co-existence also lowers the surface O_3_, NOx, and SO_2_ by 4–5% due to the heterogeneous chemistry between biomass burning and mineral dust as indicated by model simulations.

## Introduction

Biomass burning refers to natural or manmade fires with combustion of organic matter such as forest, savanna, peatland, and agricultural residual. Most of the intensive biomass burning activities are initiated in densely vegetated areas or cropland^1^. Wind-blown dust is generated from desert, arid or semi-arid areas with sparsely vegetated ground surface. Biomass burning and wind-blown dust are both closely seasonal-dependent and they usually occur in different locations and seasons^2^, thus there is low chance for them to meet with each other. In East Asia, however, biomass burning from the Peninsular Southeast Asia (PSEA) and wind-blown dust from Taklamakan and Gobi Desert (TGD) can reach to the west Pacific region over the coastal area of southeastern China and Taiwan simultaneously in boreal spring, which may cause severe air pollution due to their combining effect^3^.

Biomass burning in PSEA is predominately initiated by human activities including deforestation fire and agricultural residual burning^4^. It is generated at Cambodia, Laos, Myanmar, Vietnam, and north part of Thailand starting from late February or early March to late April or early May with the onset of East Asia monsoon, which brings intensive precipitation to the Indochina area. PSEA biomass burning occurs on the southeastern side of the Tibet Plateau and is uplifted by both the lee-side trough and topography elevation over Yungui Plateau up to 6 km height above ground surface^5,6^, where it travels to the west Pacific region before gradually disperses in the free troposphere. Although majority of biomass burning plume remains in the upper air during the long-range transport, local air qualities along the trajectory (PSEA area, south part of China, Hong Kong, and Taiwan) may get severely deteriorated during intensive burning periods^7,8^. Driven by the Mongolian Cyclone and the East Asia Trough, the TGD dust also starts in early spring over the Tarim Basin, Mongolia, and north part of China^2^. Part of the wind-blown dust is transported eastward by prevailing winds to Japan, the North Pacific^9^, and even trans-pacific to west coast of North America, while the rest is carried by the northwesterly cold fronts along the east coast of China passing through Beijing, Shanghai, and finally reaches Taiwan^10^ and significantly increases the PM_10_ concentrations along the transport pathway^11^.

Although many research efforts have been devoted to investigate the characteristics of biomass burning or dust regarding their seasonality, transport, and impacts on air quality^12–15^, very limited attention has been paid to the co-existence condition that they mix with each other. A few campaign and modeling studies^16–18^ have noticed the co-existence of Sahara dust and biomass burning smoke at the Cape Verde over western Africa and these pilot research activities focused on characterizing their aerosol optical properties. Yen *et al*.^3^ indicated that the PSEA biomass burning may be brought from 700–850hPa down to surface by the subsidence of cold surge anticyclone between 115°E and 120°E, but the chemical interactions between biomass burning gases and dust particles, and the impact on air quality under the mixing conditions were not thoroughly investigated. Unlike Cape Verde with limited urban areas, western Pacific region hosts many densely populated cities in southeast coast of China and Taiwan where the air pollution plays important role in public health. In addition, heterogeneous reactions between biomass burning and mineral dust may alter the atmospheric chemistry over the western Pacific region as well. The biomass burning plume from PSEA contains substantial amount of reactive gases including ozone (O_3_) and nitrous oxide (NOx), while mineral dust from TGD is mostly calcite, kaolinite, hematite, quartz, and crustal irons. Thus the mixing with TGD dust may not only intensify the impact of biomass burning on ground level air quality, but also alter the budgets of reactive gases through heterogeneous chemistry on the surface of dust particles and lead to production of new secondary inorganic aerosols^19^. In this study, we reveal the simultaneous presences of two different plumes and the descending procedure of biomass burning through a case study, we then analyze the intensified influence of biomass burning and dust storm on air quality for 5 years from 2006 to 2010, and finally demonstrate the changes of reactive gases due to the heterogeneous chemistry between biomass burning and mineral dust. This is the first integrated study to investigate the co-existence of biomass burning and dust in the west Pacific region by applying multiple dataset including NASA Micro-Pulse Lidar Network (MPLNET) measurement, MODIS satellite product, surface observations, and Weather Research and Forecasting/Community Multi-scale Air Quality (WRF/CMAQ) model simulations. Due to the availability of observation data, especially the Lidar data that is essential to demonstrate the vertical profile of suspended aerosols, the analysis of co-existence condition was mainly performed over Taiwan. The results of this study can help to improve our understanding of the severe air pollution induced by long-range transported biomass burning and wind-blown dust, and provide the first insight into the heterogeneous reactions between them, which could be supportive for the future local air quality management at the areas in the eastern Asia or other continents. The revealed descending procedure of biomass burning driven by onset of dust storm can also help the research community to investigate the potential impact of iron and nutrition depositions into the ocean ecosystem in the subtropical west Pacific as well.

## Results

### Identify the co-existence of PSEA biomass burning and TGD dust

**Figure 1:**
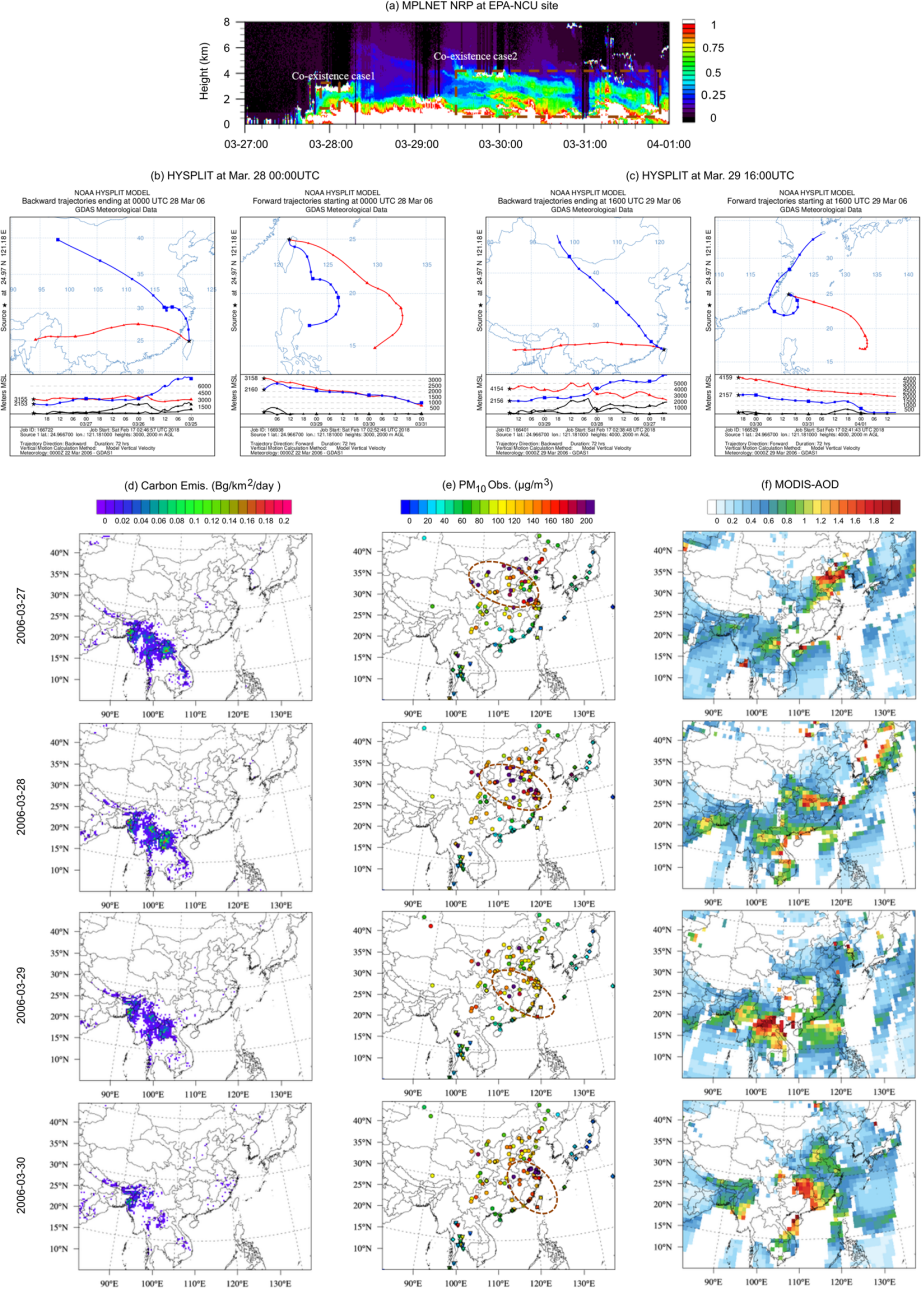
(**a**) MPLNET Lidar observation of NRB at EPA-NCU site from Mar.27 00:00UTC to Apr.01 00:00UTC 2006, the brown dashed boxes indicated the two co-existence cases; HYSPLIT backward (left side) and forward (right side) trajectory analysis at (**b**) Mar.28 00:00UTC; and (**c**) Mar.29 16:00UTC; (**d**) carbon emission from FLABME; (**e**) PM_10_ observation from API, PCD, EANET, and TAQMN; and (**f**) MODIS AOD. [Maps were made using the NCAR Command Language v6.4.0 software, http://dx.doi.org/10.5065/D6WD3XH5. HYSPLIT trajectory figures were made with the NOAA Air Resource Lab HYSPLIT online tool, https://ready.arl.noaa.gov/HYSPLIT_traj.php].

Co-existence of PSEA biomass burning and TGD dust was firstly identified based on the analysis of MPLNET monitoring data^20^ at the Taiwan Environmental Protection Administration and National Central University (EPA-NCU) site (24.97°N, 121.18°E). Vertical distribution of the Normalized Relative Backscatter (NRB) coefficient from Lidar measurement is used to demonstrate the distinct aerosol layers of biomass burning and dust. The NRB data showed a strong aerosol signal around Mar.28 00:00 UTC between 2 km and 3 km height, and, three separate layers of signals on Mar.29 16:00 UTC centering at 0.5 km, 2.5 km, and 4 km respectively as presented in Fig. 1(a). The EPA-NCU monitoring station located inside the campus of NCU and it was only 2 km from the Taoyuan City downtown area and close to two highways as well, so the near-surface layer signal between 0–1 km was primarily corresponding to the anthropogenic aerosols from local and southeast China sources^21^. Both backward- and forward-trajectory analysis with HYSPLIT^22,23^ were conducted to track the original sources of the aerosol layer at 2 km and 3 km on Mar.28 and the two aerosol layers at 2 km and 4 km on Mar.29 respectively, and also to track their movements afterwards as well, as shown in Fig. 1(b,c) respectively. The HYSPLIT backward trajectories suggested that the NRB signals at 3 km on Mar.28 and 4 km on Mar.29 were both attributed to the air plume travelled from PSEA (red line with triangle markers), and signals at 2 km corresponded to the plumes travelled from Gobi Desert (blue lines with rectangle markers). The forward trajectories suggested that plumes travelled from PSEA rapidly descended after onset of Taiwan. We then applied the Fire Locating and Modeling of Burning Emissions (FLAMBE) biomass burning emission inventory^4^ and surface PM_10_ observations to demonstrate the presence of biomass burning and dust in the sources regions and along the transport trajectories indicated by HYSPLIT. As shown in Fig. 1(d), FLAMBE suggested significant amount of biomass burning emission on Mar.27–29 over PSEA, which declined rapidly on Mar.30. The three days (Mar.27–29) intensive burning corresponded to the strong MPLNET signal at 4 km height at EPA-NCU site which also lasted for three days (Mar.29–31) and was about 2 days lagged behind because of the traveling time from PSEA to Taiwan. Figure 1(e) presents the daily PM_10_ observations from Air Pollution Index (API), Acid Deposition Monitoring Network in East Asia (EANET), Taiwan Air Quality Monitoring Network (TAQMNN; only two sites are shown here to represent the north and south regions of Taiwan due to limited figure size, full picture of PM_10_ concentrations at all the 77 TAQMN sites are shown in Fig. S1), and Thailand Pollution Control Department (PCD) networks during the same time period. The surface PM_10_ measurements suggested that the dust plume (indicated by orange dash circles) moved from Gobi Desert on Mar.27 toward southeast direction, which gradually reached central China on Mar.28, and then arrived at the southeast coast on Mar.29. Surface PM_10_ concentrations were increased substantially along the dust transport pathway as suggested by the observations from different networks. Figure 1(f) shows daily MODIS AOD for the same time period. As the daily MODIS AOD product contained a lot missing values due to the contamination by cloud, the movements of PSEA biomass burning plume and the TGD dust can be roughly visualized along the trajectories indicated in Fig. 1(b,c). The FLAMBE carbon emission and PM_10_ observation agreed well with the MODIS AOD product in terms of the spatial distribution patterns of aerosol over the studying domain, which was also consistent with the HYPLIT trajectories as well.

### Descending procedure of PSEA biomass burning

**Figure 2:**
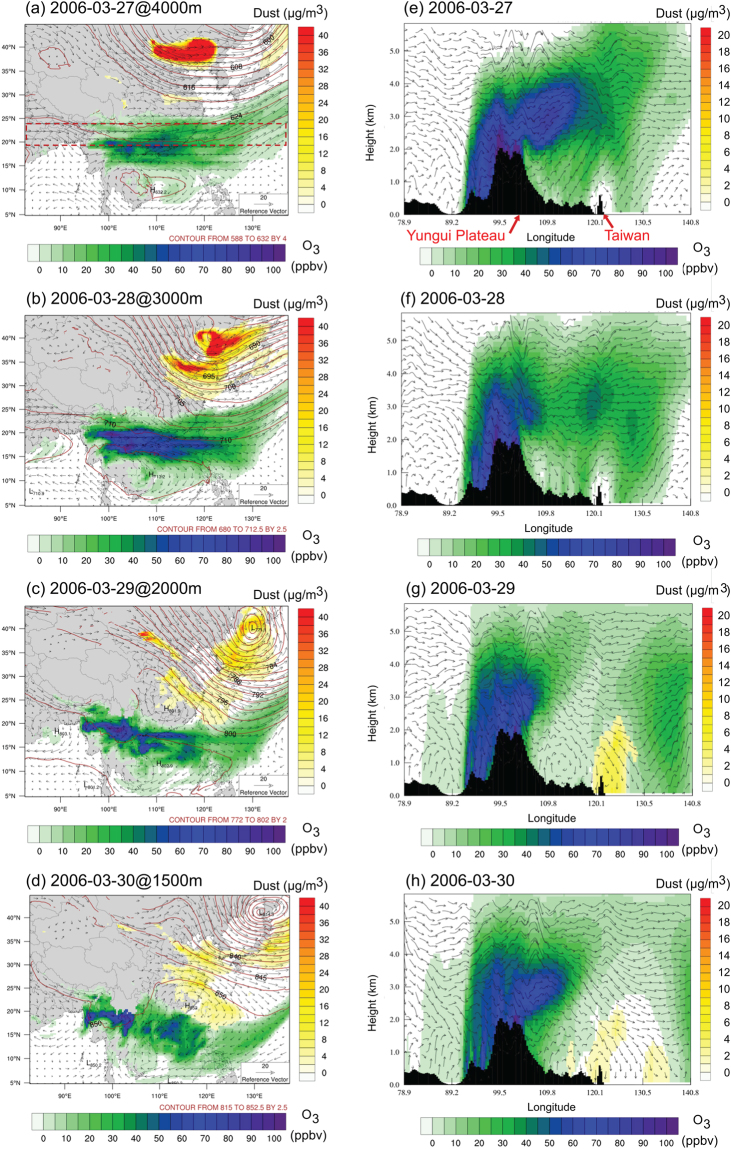
Spatial distributions of wind vector, pressure contour, dust concentration, and biomass burning O_3_ concentration on (**a**) Mar.27 at 4000 m; (**b**) Mar.28 at 3000 m; (**c**) Mar.29 at 2000m; and (**d**) Mar.30 at 1500 m above sea level; Zonal average (19°N-24°N, along the red dashed box in (**a**)) cross section distributions of wind vector, dust concentration, and biomass burning O_3_ concentration on (**e**) Mar.27; (**f**) Mar.28; (**g**) Mar.29; and (**h**) Mar.30. [Maps were made using the NCAR Command Language v6.4.0 software, http://dx.doi.org/10.5065/D6WD3XH5].

Under the co-existing condition from Mar.28 to Mar.31, 2006, the synoptic upper level trough at 700hPa which carried the subsiding TGD dust storms also drove the biomass burning plume to descend from 3–5 km in the upper air down to 1–2 km after the two plumes meet together as shown in Fig. 2. The freshly generated dust plume can be bought up to 6 km above the ground at source region over northwest China^2^, and it gradually descended with the large scale subsidence along the southeastward transport^10^. Figure 2 presents the spatial distributions of TGD dust, PSEA biomass burning O_3_, and the meteorology simulated with WRF/CMAQ for the co-existence case on Mar.28–30, 2006. On Mar.27, both TGD dust and PSEA biomass burning at 4 km above sea level were carried by the Westerlies but at different longitudes towards east as shown in Fig. 2(a). Meanwhile, the cross-section distributions of biomass burning O_3_ (along the red dashed rectangles between 19°N −24°N shown in Fig. 2(a)) and wind direction presented in Fig. 2(e) revealed the upward movement of biomass burning plume from 100°E to 120°E due to the lee-side trough and topographic elevation on the eastern side of Yungui Plateau (23–28°N, 100–105°E). The WRF/CMAQ simulation is well consistent with the HYSPLIT backward trajectories shown in Fig. 1(b,c) which demonstrated the topographic elevation of biomass burning plume, fairly consistent with the WRF/CMAQ simulations. On Mar.28, part of the dust plume started to move southeastward driven by the upper level trough at 3 km (~700hPa) along the eastern coast of China as shown in Fig. 2(b), but the PSEA biomass burning was not affected yet as shown in Fig. 2(f). On Mar.29, the upper level trough grew deeper at 2 km (~800hPa) with the formation of low pressure anticyclone centering over the Sea of Japan, which drove the TGD dust move further southeast towards Taiwan and pushed the PSEA biomass burning to lower latitude from 25°N to 20°N as shown in Fig. 2(c). Meanwhile, the PSEA biomass burning plume was also pushed downward between 110°E and 120°E over Taiwan by the large scale subsidence as shown in Fig. 2(g). The prevailing southeast wind lasted for about 2 days until the subtropical high center moved from South China Sea to mainland China (at Fujian Province) on Mar.30 as shown in Fig. 2(d), which pushed the TGD dust plume follow the clock-wise cyclone over East China Sea before reaching Taiwan. On one hand, TGD dust increased the local PM_10_ concentrations along the pathway during the southeastward travelling period (shown in Fig. 1(d)) and landed at north part of Taiwan below 2 km height. One the other hand, the PSEA biomass burning plume stayed in the free troposphere at 4−6 km height before reaching Taiwan, but rapidly descended to the near-surface layer with the onset of TGD dust. The dynamic movements of biomass burning also explained the surface PM_10_ changes shown in Fig. 1(e). Although the MODIS product (Fig. 1(e)) suggested high AOD along the biomass burning transport pathway (~20°N) over Guangxi, Guangdong, and Hainan provinces of China, the surface PM_10_ concentrations in these areas were barely affected during the intensive burning period because there was no significant intrusion from upper air biomass burning plume to the ground surface. The surface PM_10_ at Hong Kong and Taiwan however, were severely enhanced due to the subsiding of biomass burning. The dynamics of dust and biomass burning mixing over the western Pacific shown in this study is completely different from the meteorology conditions controlling the mixing of dust and biomass burning over the western Africa. Sahara dust is elevated and transported southwestward towards Atlantic from northern western Africa by low-level east-northeasterly flow below 1.5 km above sea level, and biomass burning from southern western Africa is frequently brought to 2.5−4 km by deep convections while it travels northwestward towards Atlantic^24^. Thus the warm air laden with biomass burning tends to be lifted to even higher altitudes as it overrides the cooler, drier dust laden air, and the near surface air at western Africa does not receive significant impact by the upper air biomass burning.

### Severe air pollution under the co-existence condition

**Figure 3:**
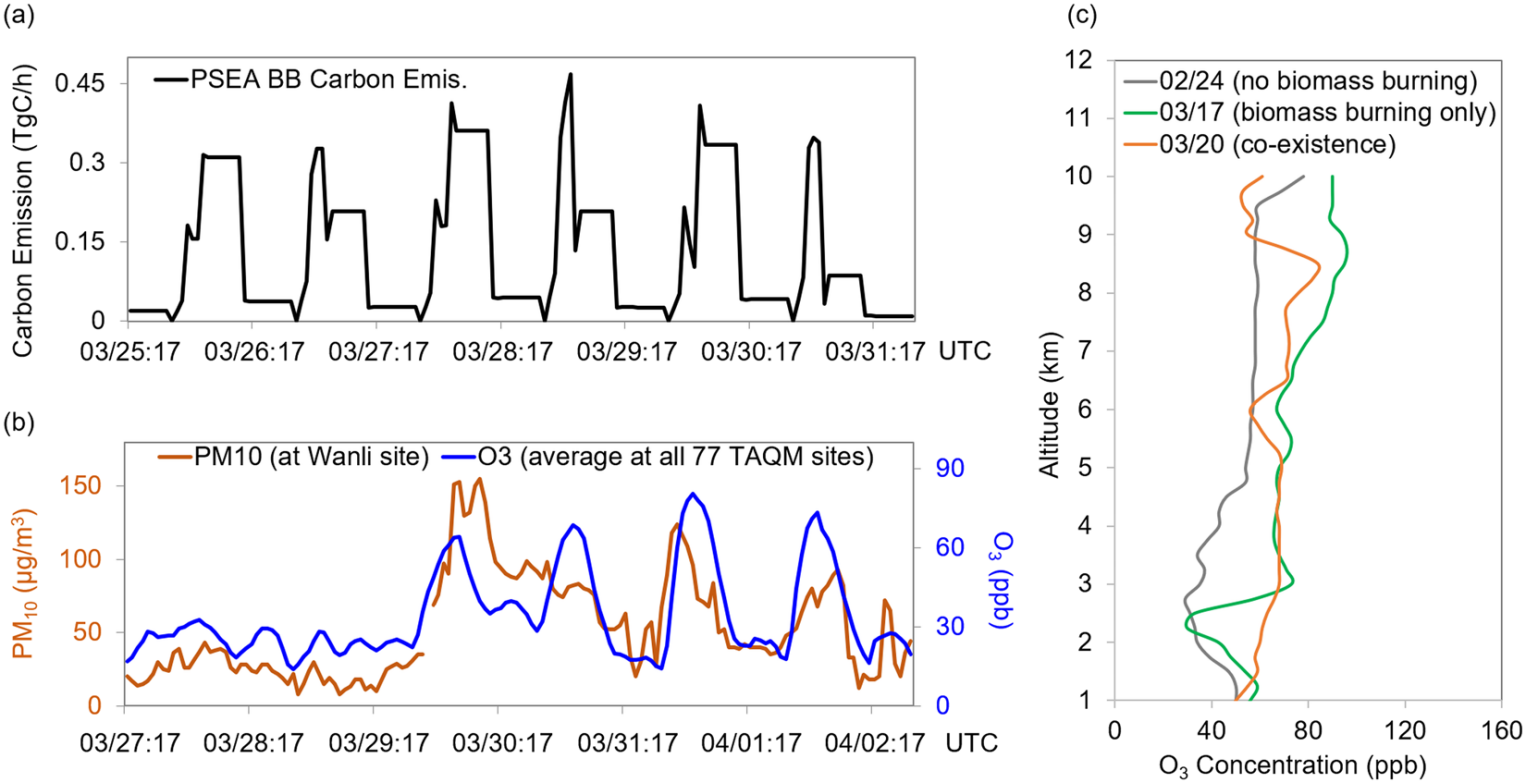
Hourly changes of (**a**). PSEA biomass burning carbon emission (TgC) and (**b**). O_3_ (ppb) observations averaged at all 77 TAQMN sites and PM_10_ measurements (µg/m^3^) at Wanli site; (**c**). Sounding measurements of O_3_ vertical profiles on Feb.24 (grey line), Mar.17 (green line), and Mar.20 (orange line) representing the cases of no biomass burning, biomass burning only, and co-existence condition. [The figure was made with Office365-Excel, Microsoft, Redmond, WA, USA].

Some pilot studies have evaluated the impact of PSEA biomass burning or TGD dust on local air quality over Taiwan^5,8,12,25–27^, but few of them have noticed that the co-existence condition may cause extremely polluted air pollution^28^. Although the research community noticed that the observed concentrations of air pollutants in boreal spring over Taiwan are about 3 times higher than that in other seasons^26^, most of the previous impact assessments tried to attribute the deterioration of air quality to either one of the sources only because biomass burning impacts were usually evaluated on monthly or seasonal scale while dust storm lasted for only 2–3 days. For example, Lin *et al*.^29^ reported that the dust storm in Mar.18 2005 resulted in peak CO concentration of 1.0ppm at a background station and attributed it to the contribution of anthropogenic pollutants from Asia continent carried along by the dust storm. But long-range transport from Asia continent was estimated to contribute only 230ppb of CO during winter monsoon periods^30^. While anthropogenic CO emission at southeast China usually peaks in winter due to fossil fuel combustion for heating^31^, the excessive CO measured at background site in March was more likely due to the influence from biomass burning too. To demonstrate how dust storm intensifies the influence of biomass burning and deteriorates the local air quality, hourly changes of PSEA biomass burning emission and the surface observations of PM_10_ and O_3_ at Taiwan were investigated for Mar.27-Apr.02 2006 as presented in Fig. 3. Since the PSEA biomass burning plume took about 2 days traveling to Taiwan, the biomass burning carbon emission were presented 48 hours prior to Taiwan surface observations. Surface PM_10_ measurements were collected from the Wanli site (25.18°N, 121.69°E) to indicate the onset of dust storm. Wanli is a background station away from the local anthropogenic emission sources. Observations from the Wanli site is frequently used as an indicator to identify the onset of TGD dust storm^10^ when the measured PM_10_ concentration exceeds 100 µg/m^3^. Surface O_3_ were averaged from the observations at all TAQMN sites to demonstrate the domain-wide impact of biomass burning over Taiwan. As presented in Fig. 3(a), the PSEA biomass burning showed relatively stable daily carbon emission with prominent diurnal cycle from Mar.23 to Mar.29, 2006. The temporal changes of surface O_3_ however, were not exactly following the trend of biomass burning emission as demonstrated by the low concentrations (<30ppb) from Mar.27 to Mar.29. Instead, variations of O_3_ showed very similar pattern as the changes of PM_10_ concentration at Wanli site, and high O_3_ peaks were almost always associated with the incoming of dust. With the onset of dust on Mar.29, O_3_ concentration over Taiwan was rapidly increased from less than 60 ppb to more than 100 ppb within 12 hours, indicating the important intensified influence of biomass burning on surface air quality under the co-existence condition. We also collected sounding data at Taipei (25.02°N, 121.50°E) to demonstrate the O_3_ vertical profiles under the influences of biomass burning and dust storm as shown in Fig. 3(c). As compared to no biomass burning case (Feb.24, 2006), O_3_ concentration under biomass burning only case (Mar.17, 2006) was generally in the same level below 2 km, but rapidly increased from 2 km to 3 km and was 30–40ppb higher within 2–5 km height due to the contribution of PSEA biomass burning^11^. Under the co-existence case (Mar.20, 2006) however, O_3_ concentration was significantly higher than the other two cases starting from 1.5 km height. The O_3_ vertical profiles from sounding measurements were consistent with the demonstration from Fig. 2, suggesting that the dust storm brought excessive biomass burning O_3_ from the upper air towards near surface layer.

### The intensified air pollution under the co-existence conditions during the 5 years studying period

**Figure 4:**
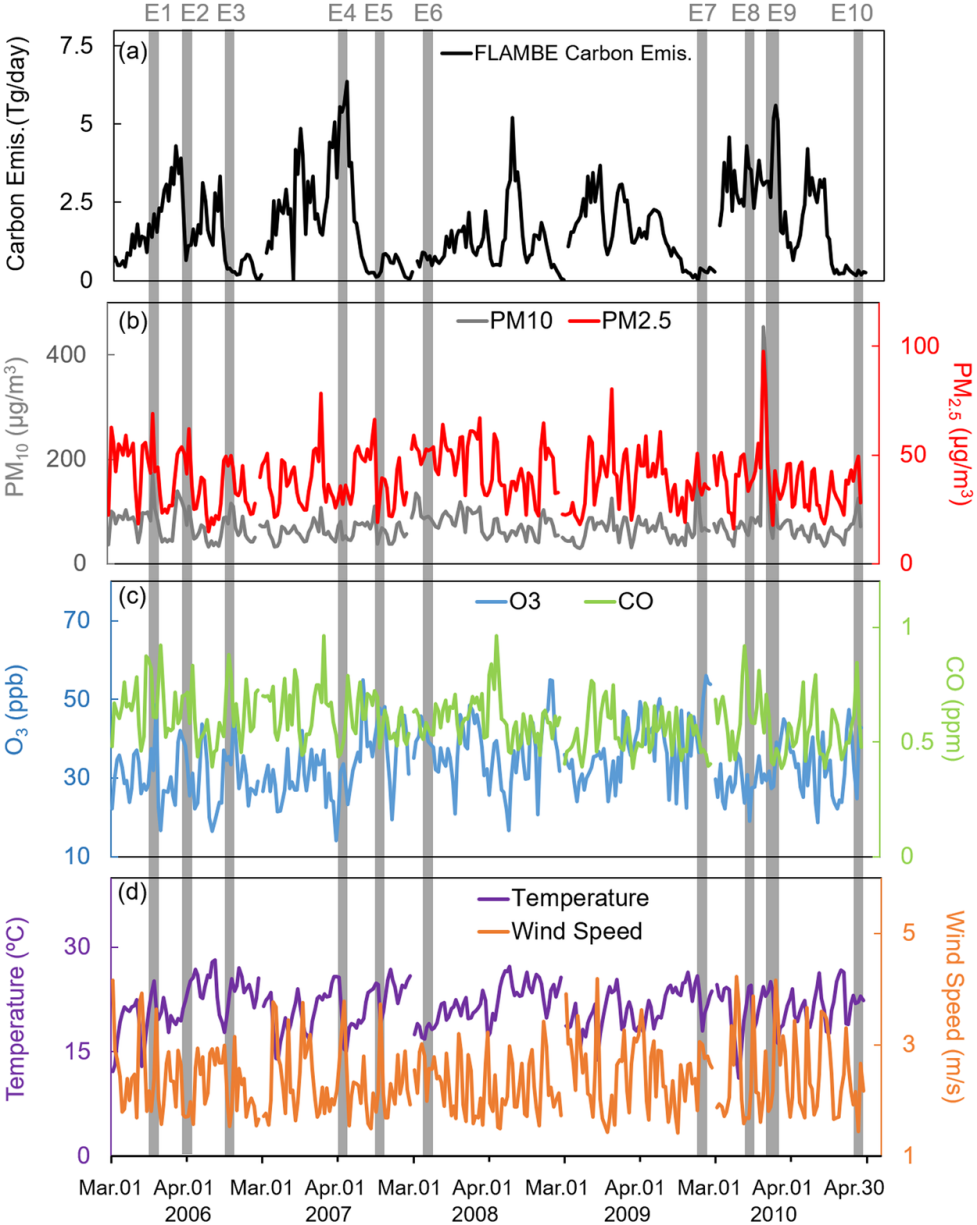
Daily variations of (**a**). PSEA biomass burning carbon emission (TgC), surface concentrations of (**b**). PM_10_, PM_2.5_, (**c**) O_3_ and CO, and (**d**). temperature and wind speed averaged at all TAQMN sites. [The figure was made with Office365-Excel, Microsoft, Redmond, WA, USA].

Figure 4 presents the daily carbon emission from PSEA biomass burning, surface measurements of PM_10_, PM_2.5_, O_3_, CO, temperature, and wind speed averaged at all TAQMN observation sites during March and April from 2006 to 2010 (biomass burning emission is presented two days prior to the surface observations in Taiwan to accommodate for the traveling time). As indicated by the dust storm records from Taiwan EPA, a total of 10 dust storm events were identified during this period (E1-E10, indicated by grey boxes in Fig. 4), which were all treated as co-existence events in this study due to the relatively persistent existence of biomass burning in boreal spring. During event E3, E5, E6, E7, and E10, although the PSEA biomass burning emissions were substantially lower than the other days, the surface concentrations of O_3_ and CO pollutants were significantly higher, indicating that the intrusion of biomass burning from the upper air severely affect the surface air quality. During the event E3 for example, surface concentrations of O_3_ and CO were increased from 33ppb and 0.56ppm on Apr.19 to 46ppb and 0.79ppm respectively on Apr.20–21. Meanwhile, carbon emission from PSEA biomass burning was decreased from 0.41TgC/day on Apr.17 to 0.27TgC/day on Apr.18. The comparison of the O_3_ and CO before and during the co-existence events suggest that higher PSEA biomass burning emission may not necessarily lead to more significant impact on the surface air quality in downwind receptor areas. Instead, the descending of biomass burning from the upper air driven by onset of dust storm plays a more important role in determining the influence of the long-range transport biomass burning. As compared to the no-dust events episodes during the study period, air pollutants concentrations under co-existence events are 69%, 37%, 20%, and 18% higher for PM_10_, PM_2.5_, O_3_, and CO respectively as demonstrated by the TAQMN observations.

### Heterogeneous chemistry between dust and biomass burning

**Figure 5:**
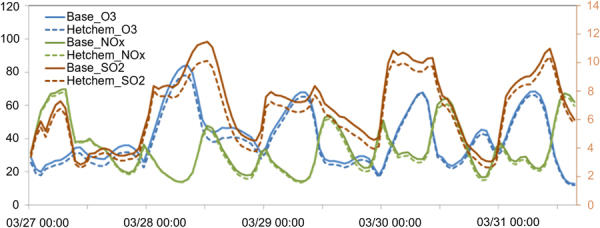
WRF/CMAQ simulated temporal changes of O_3_, NOx (using primary Y-axis on the left side), and SO_2_ (using secondary Y-axis on the right side) concentrations with (dash lines) and without the dust heterogeneous chemistry. [The figure was made with Office365-Excel, Microsoft, Redmond, WA, USA].

The reactive gases (O_3_, SO_2_, and NOx) carried by biomass burning plume can adsorb onto the surface of suspended particles in the presence of water vapor. Since TGD dust is usually carried by subsiding cold frontals, co-existence condition may intensify the heterogeneous reactions as the plume descending procedure favors the condensation of water vapor on the surface of excessive dust particles. Figure 5 demonstrates the changes of O_3_, NOx, SO_2_ during the Mar.28–31 co-existence event (E1 shown in Fig. 4) simulated by the WRF/CMAQ model with (solid lines) and without (dash lines) the dust heterogeneous chemistry^11^. Concentrations of O_3_, NOx, and SO_2_ were rapidly reduced by up to 6ppb, 2ppb, and 1.5ppb respectively within a couple of hours after the onset of dust. On five years average (2006–2010), heterogeneous chemistry between biomass burning and dust helped to lower the monthly average concentrations of O_3_, NOx, and SO_2_ by 2ppb(4%), 1.5ppb(5%), and 0.3ppb(5%) respectively.

## Discussion

The analysis conducted based on multiple observations and model simulations in this study demonstrated the descending procedure of biomass burning induced by onset of dust storm and the subsequent air pollution effects under the co-existence condition. The availability of observations especially the Lidar data, however, still limit our further investigation of the co-existence events in terms of their spatial distribution and impact on regional climate. The MPLNET measurement at NCU site is the only aerosol vertical distribution data that is accessible to demonstrate the structures of different aerosol layers in the west Pacific region. While model simulations shown in Fig. 2 indicated that co-existence may also occur over southeast coastal areas of China such as Guangdong and Fujian provinces, no Lidar observation is available to validate the simulation. Although satellite product such as CALIPSO also gives a glance of the vertical distributions of aerosols, it’s relatively too coarse in terms of both the vertical resolution and the temporal coverage as co-existence usually last for only 2 or 3 days at around 1.5–3 km height. Since the co-existence condition considerably affect the surface air pollutants as demonstrated in this study, more Lidar data and aerosol measurements are in urgent need for further evaluate the impacts of biomass burning and dust as it is of special importance to the local air quality management over southeast China and Taiwan. In addition, unique tracers such as levoglucosan for biomass burning and aluminium mass content in coarse particles for dust would be better indicators than O3 and PM10 used in this study. Measuring levoglucosan and aluminium are also recommended for future studies as it will greatly benefit the understanding of biomass burning and dust with observational method.

A more thorough investigation the co-existence is important for improving the understanding of not only the air pollution but also the regional climate change in the west Pacific region. Vertical distributions of aerosols, especially relative to the cloud height, predominately determine the aerosol radiative forcing effects. With excessive biomass burning aerosols descended from free troposphere to surface layer, the vertical temperature profiles and cloud activities could be altered which subsequently change the convective precipitation as well. The heterogeneous reactions between reactive gases and mineral dust will also lead to production of new SIA particles, which may change the aerosol characteristics in terms of number and size distribution, hydrophobic capacity, extinction coefficient, and core-shell mixing status which are all remaining unknown due to limited understanding and observations of the mixed dust and biomass burning aerosols. Pilot studies have suggested that aged biomass burning particles may have completely opposite radiative forcing property as compared to the fresh generated particles^16^, and the acidified dust particles may mobilize the dissolution ratio of iron and others and subsequently affect the rate of carbon fixation in high-nutrient and low-chlorophyll regions of the Pacific Ocean^32^. The characteristics changes of suspended particles, especially for biomass burning and dust in the upper air over the west Pacific region, may play an important role in the regional climate by altering the radiative forcing budget, cloud formation and lifetime changes, and precipitation as well. Moreover, aerosol depositions are enhanced under the co-existence condition since the suspended biomass burning plume is down-dragged to surface layer by the dust storm, yet the intensified impact on ocean ecosystem hasn’t been evaluated. Excessive organic carbons from biomass burning and minerals from dust deposited into the west Pacific may severely alter the oceanic primary productivity^33,34^.

Co-existence condition differs from the individual long-range transport of biomass burning or dust and it has unique characteristics. The results from this study demonstrate the procedures of descending biomass burning from free troposphere down to the surface air and meet with wind-blown dust, and also reveal the related severe air quality impact. Asia dust storms gradually decline since 1970s according to the record from China over the desert area^2^. Although some studies^35,36^ suggested that the earlier vegetation green-up may have reduced the dust storm frequency, the research community has no solid conclusion of the determining factor(s) of dust climatology. And the large scale drought over Asia resulted in severe dust storms in 2010^37^, indicating the extreme dust storm events could still occur in the presence of favorable meteorology condition. Meanwhile, biomass burning in Southeast Asia showed moderate annual variation from 1997–2017 but no prominent decreasing or increasing trend was found^38^. It’s important to notice that even no dust storms were reported over Taiwan after 2013, the frontal systems would still push the biomass burning plume from upper air down to the surface and deteriorate the air quality. Thus the dynamics of descending biomass burning plume demonstrated in this study can serve as an informative reference for future studies analyzing the biomass burning effect during heavy polluted episodes.

## Methods

We used multiple datasets from observations and model simulations to investigate the co-existence of biomass burning and dust in this study. Lidar data was used to identify the presences of multiple aerosol layers at different height. The MPLNET Lidar system uses laser light to measure the amount of light backscattered by atmospheric molecules, aerosols and clouds by up to 20 km above surface ground. The EPA-NCU MPLNET site provide hourly records of observations starting from 2005 but not for everyday due to instrument maintenance. HYSPLIT trajectory analyses were conducted with the NCEP Global DATA Assimilation System (GDAS) archived data at the time points indicated by the MPLNET data. Backward trajectories were used to help understand the original sources of upper air aerosols over the EPA-NCU site, and forward trajectories were used to demonstrate the descending procedures of biomass burning plume. The ground surface PM_10_ observations from 86 API sites (http://datacenter.mep.gov.cn), 11 EANET sites (http://www.eanet.asia/), 25 PCD sites (http://www.pcd.go.th/indexEng.cfm), and 77 TAQMN sites were used together with the FLAMBE biomass burning emission data and MODIS AOD product to demonstrate the spatial distribution and movement of PSEA biomass burning and TGD dust. Surface measurements and MODIS product were collected on daily scale. Sounding measurements of O_3_ were collected from Central Weather Bureau (CWB) for 2006 at Taipei to demonstrate the different vertical profiles of O_3_ under no biomass burning, biomass burning only, and coexistence conditions. Sounding data was collected on daily scale with vertical resolution of 0.25 km but only 2–3 measurements were conducted for each month. We then analyzed the meteorology field, dust and biomass burning O_3_ concentrations simulated with the WRF/CMAQ for this co-existence case to reveal the atmospheric dynamics during the mixing procedure. And finally we evaluated the impact of co-existence on air quality based on TAQMN observation data, and estimate the changes of atmospheric chemistry due to the heterogeneous reactions between biomass burning and dust over Taiwan.

For WRF/CMAQ model simulation, we used the FLAMB emission inventory to configure the biomass burning emission based on previous studies which validated the accuracy and uncertainties of multiple biomass burning emission inventories^12,39^. Dust plume rise scheme was revised based on the default CMAQv5.0.1 scheme, with the newly implemented heterogeneous chemistry validated in previous study^11^. WRF/CMAQ model simulations were conducted at 36 × 36 km grid resolution over East Asia for March and April from 2006 to 2010 with through validation performed^8^. We designed and conducted different sets of simulations with brute-force method to estimate the impact of a certain variable. These simulations include:^40^. BASE: simulation without biomass burning emission, dust plume rise or heterogeneous chemistry; (2). BIOM: simulation same as BASE but include the FLAMBE emission; (3). DUST: simulation same as BIOM but include dust plume rise scheme; and (4). CHEM: simulation same as DUST but include heterogeneous chemistry. Contributions of biomass burning O_3_ was estimated based on the difference between BIOM and BASE; contributions of TGD dust was estimated based on the difference between DUST and BIOM; contributions of co-existence was estimated based on the difference between DUST and BASE; and impact of heterogeneous chemistry was estimated based on the difference between CHEM and DUST.

## Electronic supplementary material

Supplementary Information
