# Supplementary material for: Analysis of the Co-existence of Long-range Transport Biomass Burning and Dust in the Subtropical West Pacific Region
Source: Sci Rep. 2018 Jun 12;8:8962. doi: 10.1038/s41598-018-27129-2 (PMC5997683; doi:10.1038/s41598-018-27129-2)
Supplement: Supplementary file 1 — Supplementary Information [file 41598_2018_27129_MOESM1_ESM.pdf]

Analysis of the Co-existence of Long-range Transport Biomass Burning and Dust in the Subtropical West Pacific Region

Xinyi Dong[1], Joshua S. Fu[1,2,*], Kan Huang[1,3], Neng-Huei Lin[4], Sheng-Hsiang Wang[4], Cheng-En Yang[1]

[1]Department of Civil and Environmental Engineering, the University of Tennessee, Knoxville, TN 37996, USA

[2]Climate Change Science Institute, Oak Ridge National Laboratory, Oak Ridge, TN 37831, USA

[3]Center for Atmospheric Chemistry Study, Department of Environmental Science and Engineering, Fudan University, Shanghai 200433, China

[4]Department of Atmospheric Sciences, National Central University, Chung−Li 32056, Taiwan

[*] Correspondence to: Joshua S. Fu (jsfu@utk.edu)

Fig.S1 demonstrated the daily observed PM_{10} and O_3 concentrations at TAQMN sites. With onset of dust, rapid enhancement of PM_{10} concentrations was found all over Taiwan from Mar.28 to Mar.29, 2006, indicating the significant impact of long-range transported dust from the Taklimakan and Gobi Desert. Meanwhile, O_3 concentrations were also elevated during the same period, suggesting that the impact of biomass burning was intensified under the co-existence condition.

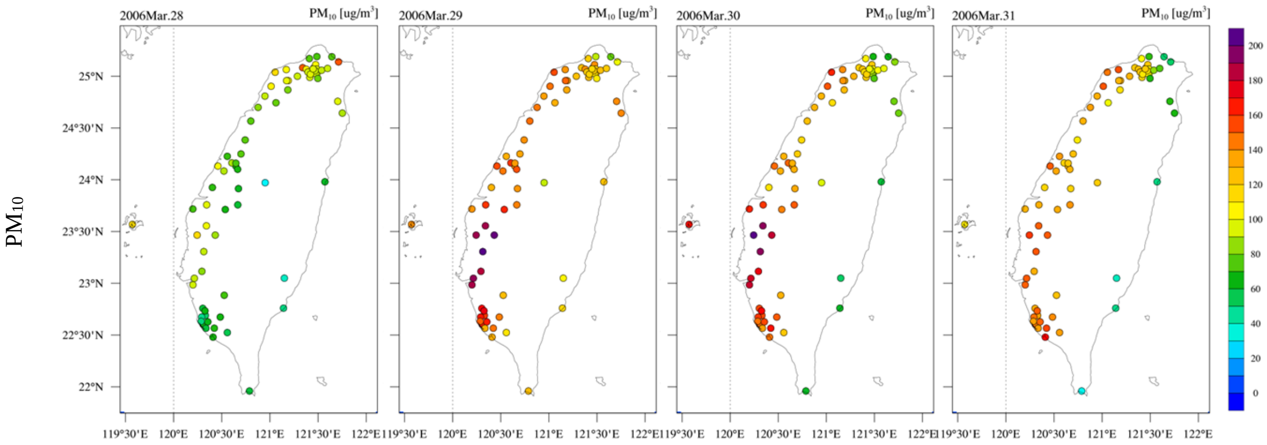

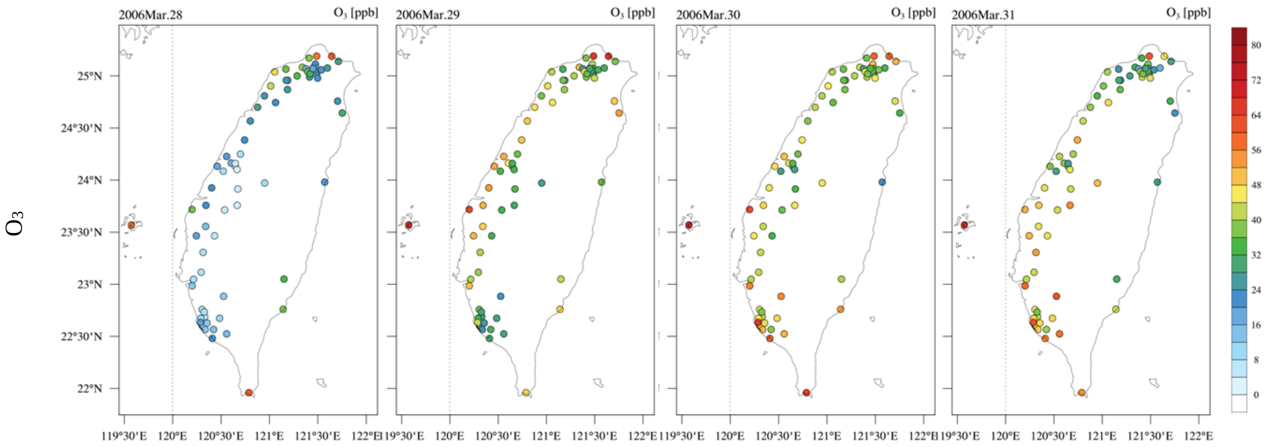

Figure S1. Observed PM_{10} and O_3 concentrations at all 77 TAQMN sites during Mar.28-31, 2006.
